# Genetic Variation in Disease Resistance Against White Spot Syndrome Virus (WSSV) in *Liptopenaeus vannamei*

**DOI:** 10.3389/fgene.2019.00264

**Published:** 2019-03-28

**Authors:** Trinh Thi Trang, Nguyen Huu Hung, Nguyen Huu Ninh, Wayne Knibb, Nguyen Hong Nguyen

**Affiliations:** ^1^Faculty of Science, Health, Education and engineering, GeneCology Research Centre, University of the Sunshine Coast, Maroochydore, QLD, Australia; ^2^Vietnam National University of Agriculture, Gia Lâm, Vietnam; ^3^Research Institute for Aquaculture No. 3, Nha Trang, Vietnam

**Keywords:** heritability, correlations, selective breeding, genetic improvement, viral resistance, shrimp

## Abstract

White Spot Syndrome Virus (WSSV) is the most damaging pathogen in terms of production and economic losses for the shrimp sector world-wide. Estimation of heritability for WSSV resistance was made in this study to obtain necessary parameter inputs for broadening the breeding objectives of an ongoing selective breeding programme for Whiteleg shrimp (*Liptopenaeus vannamei*) that has focussed exclusively on improving growth performance since 2014. The present study involved a disease challenge test experiment using a total of 15,000 shrimps from 150 full- and half-sib families (100 individuals per family). Survival rates were recorded at six different experimental periods: 1–3 days (S1), 1–5 days (S2), 1–7 days (S3), 1–9 days (S4), 1–12 days (S5), and 1–15 days (S6) and were used as measures of WSSV resistance. There was significant variation in WSSV resistance among families studied. Quantitative-real time PCR (qPCR) analysis showed that the amount of viral titer (viral load) was significantly lower in high than low resistance families. Analyses of heritability were carried out using linear mixed model (LMM) and threshold logistic generalized model (TLGM). Both linear and threshold models used showed that the heritability (h^2^) for WSSV resistance was moderate in the early infection phases (S1–S4), whilst a low h^2^ value was observed for survival after 12 and 15 days of the challenge test (S5 and S6). The transformed heritabilities for WSSV resistance ranged from 1 to 31% which were somewhat lower than those estimated on the liability scale. Genetic correlations between survival rates measured over six different days post-infection were high and positive (0.82–0.99). The phenotypic correlations ranged from 0.31 ± 0.01 to 0.97 ± 0.01. The genetic correlations between body weights and WSSV resistance were negative. Our results on the heritability and genetic correlations show that improvement of WSSV resistance can be achieved through selective breeding in this population of Whiteleg shrimp.

## Introduction

Shrimp culture is rapidly expanding with the current production of 4.86 million tones, contributing 6% of the total aquaculture production worldwide in 2014 (FAO, 2016). *Liptopenaeus vannamei* is the main species for the shrimp sector due to its advantageous biological features and the availability of genetically improved shrimp lines (Thitamadee et al., 2016). Nevertheless, in recent years viral diseases such as Taura syndrome virus (TSV) (Stentiford et al., 2009), white spot syndrome virus (WSSV) (Oidtmann and Stentiford, 2011), and yellow head virus (Senapin et al., 2010) have caused significant economic losses (approximately USD 6 billion in 2016) for the shrimp sector (Rizan et al., 2018). WSSV is the most adverse pathogen to shrimp farming (Escobedo-Bonilla et al., 2008; Sánchez-Paz, 2010). Infection of WSSV on Whiteleg shrimp is characterized by a rapid mortality up to 100% within 7–10 days. In grow-out culture, shrimp at all ages and sizes are sensitive to WSSV; the highest mortality rate is usually recorded in the farming period of 1–2 months after stocking (Kasornchandra et al., 1998). To date, measures to control WSSV include improving environmental rearing conditions and management practices (Rahman et al., 2007) or using specialized formulated diets to boost shrimps’ immune system (Rajkumar et al., 2017), as well using vaccines (Rijiravanich et al., 2008). However, none of these methods are cost-effective and practically feasible (Phuthaworn et al., 2016). Biosecure nuclei in the selection environment are required to meet a great demand for free-pathogen seeds to supply commercial production. Therefore, the development of high WSSV resistant lines of *L. vannamei* through conventional selective breeding approach has been initiated as a first step of a long-term genetic improvement program for this species to promote sustainable development of the shrimp sector world-wide.

Selective breeding has been applied to accelerate genetic improvement for many commercial traits of economic importance (Nguyen, 2016; Gjedrem and Rye, 2018). In *L. vannamei*, the majority of the selective breeding projects have been carried out with an emphasis on growth improvement (De Donato et al., 2005; Lu et al., 2015; Luan et al., 2015). There are also attempts to select for improved resistance to contagious diseases, such as Taura syndrome (Cock et al., 2009; Ødegård et al., 2011) or white spot syndrome (Gitterle et al., 2006b; Cuéllar-Anjel et al., 2011).

Estimations of heritability for WSSV resistance in these studies were based on laboratory challenge test experiments or on mortality loss recorded after natural outbreaks during a grow-out phase in farms (Gitterle et al., 2005, 2006b; Caballero-Zamora et al., 2015; Sui et al., 2016). To date, there is, however, no published information regarding genetic parameter estimates for WSSV resistance at different times of infection in juvenile Whiteleg shrimp. WSSV resistance at different times of infection can be genetically different traits, as it may be related to innate immune differences between the animals. Depending on the virulence of the virus, type of the challenge test as well many other factors, the innate immune response of shrimps may fluctuate with infection times. Also, genetic relationships between growth related traits and WSSV resistance are reported to vary with growth or infection stages. Caballero-Zamora et al. (2015) obtained a positive genetic correlation between the two traits at post-larval stage 10, whereas the correlation estimate was negative in a later stage of growth development (Gitterle et al., 2005; Campos-Montes et al., 2013). Knowledge on genetic variation in WSSV resistance and its relationship with body weight at different stages of growth development will provide basic information to develop genetic measures to improve commercial traits in the future breeding selection programmes for this species.

The aims of this study were to estimate the heritability for WSSV resistance and its genetic correlation with body weights during the early juvenile stage and at harvest. Additionally, the study analyzed WSSV titer to understand pathogenic differences between the high and low WSSV resistance families.

## Materials and Methods

### Genetic Population

Shrimps used in this study originated from the third generation of a Whiteleg shrimp population selected for high growth at Research Institute for Aquaculture N.3 (RIA3), in Nha Trang, Khanh Hoa, Vietnam. A detailed description of the population was given in Ninh et al. (unpublished). Briefly, the founder population included eight stocks from Mexico, Ecuador, Colombia, United States of America (three populations), Thailand, and Indonesia. In 2014, a partial diallel cross was carried out to form a base population (G0) for selection. Selection has been practiced for high growth over 3 years: 2015 (G1), 2016 (G2), and 2017 (G3). In 2017, a total of 150 full- and half-sib families were produced from which 200 representatives of each family were randomly sampled for this study.

### Family Production, Rearing, and Tagging

Breeding, rearing to tagging was based on commercial standard procedures as described in earlier studies, e.g., Argue et al. (2002) and Castillo-Juárez et al. (2007). Prior to breeding, breeders were fed a high protein brood-stock diet and fresh foods (i.e., seaworms) four times a day (6 a.m., 10 a.m., 4 p.m., and 10 p.m.). After 20 days, unilateral eyestalk of females was ablated. When breeders reached maturation stage, spermatophores of each male were collected and placed into thelycums of two female breeders to produce 150 full- and half-sib families. The female breeder was placed in an individual tank (500 liter) until spawning in 3–5 h. Eggs were then hatched in spawning tanks in 8–10 h before nauplii from each family were collected to rear separately in 500 L-tanks with the density of 150 individuals/L. Larvae were fed after 3 days post-hatching with *Chaetoceros* sp., commercial feed and then *Artemia* sp. From post-larvae 12, shrimp was moved to a new 200 L-tank with the stocking density of 50 post-larvae/L and fed with commercial feed (40% protein) four times per day. Rearing tank maintained water temperature at 26–28°C and salinity was around 31–32 ppt with 50% daily water exchange. Aeration was applied continuously to keep the level of dissolved oxygen (DO) at 5.0 ± 1.0 mg/l.

When shrimp reached an average size of 2 g at about 50–70 days post hatching, they were collected for PCR screening of four pathogens (IHHNV, YHV, TSV, and AHPNV) to ensure that the juveniles were disease-free for tagging. A total of 200 shrimps per family were then tagged on family level basis using Visible Implant Elastomer, VIE (Northwest Marine Technology). Two different colored tags among five color options (red, orange, green, yellow, and pink) were inserted at the first left and sixth right abdominal segments. Totally 30,000 shrimps from 150 families were tagged and measured for body weight and length. A total of 15,000 shrimps (100 individuals per family × 150 families) were used for the challenge test experiment (section Challenge Test) and the other half (15,000 individuals) were kept in bio-security rearing systems as a back-up population.

### Preparation for Challenge Test

The challenge test was conducted at National Centre for Marine Breeding (NCMB) of RIA3. NCMB was authorized by Department of Animal Health, Vietnam to perform infectivity experiments in aquatic animals. Shrimp diagnosed with WSSV infection was collected from grow out shrimp ponds in Ben Tre province, Vietnam and confirmed by PCR/RT-PCR for WSSV infection and other pathogenic absence (Pan et al., 2008). Histological tests were also performed by manipulation of preserved exoskeleton in Davidson’s fixative, paraffin embedding, slicing, and Hematoxylin and eosin staining (Gitterle et al., 2005).

Preparation for virus aliquots was made following the procedure of Li et al. (2013). The exoskeleton (5 g) of a frozen, naturally diseased Whiteleg shrimp that had positive WSSV diagnostic was extracted with 10 ml PBS (8 g NaCl, 0.2 g KCl, 1.44 g Na_2_HPO_4_, 0.24 g K_2_HPO_4_, pH adjusted to 7.4, and dH_2_O added to 1 L) supplemented by a vigorous vortex and centrifuged at 3000 rpm for 10 min at 4°C. The supernatant was filtered through a 0.45 μm membrane. Number of viral copies (or viral titer, viral load) was quantified using quantitative real-time PCR (qPCR).

### Challenge Test

Tagged shrimp (100 individuals/family) were acclimated 3 days prior to the challenge experiment. Water parameters were maintained under a similar condition following a standard commercial practice (temperature = 26–28°C, Salinity = 31–32 ppt, 50% daily water exchange and full air supply). The challenge test was carried out by communal rearing of families in five 10 m^3^ tanks in two replicates (10 individuals/family × 150 families per tank in each replicate) over a 15 day experimental period. Water-borne infection was applied by placing shrimp in WSSV-infected water containing viral copy concentration of 2 × 10^4^ WSSV copies/ml in the first day and then the shrimps were moved to a new tank which contained uninfected filtered seawater for the remaining experimental period. The experimental shrimps were fed with a commercial diet containing 40% protein, three times a day (8 a.m., 1 p.m., and 6 p.m.) with the feeding rate of 10% of total shrimp body mass. Temperature, pH, DO and other water parameters was kept stable and frequently monitored by a Water Quality Checker (PC 650, Eutech, Singapore). During the challenge test, strict biosecurity measures were taken, including disinfection of the outflow water, decontamination of facilities and disposal of dead shrimps (Department of Animal Health Standard).

### Real-Time PCR for Sample Analysis

To understand pathogenic differences between the high and low resistance families (mean survival = 49.5 vs. 30.1%, respectively), WSSV titer was analyzed using quantitative-real time PCR (qPCR). The moribund shrimps from the challenge test were collected and stored in Ethanol 70°. DNA from five moribund shrimps from each family (10 mg pleopods per each sample) were extracted by Qiagen Dneasy Kit. DNA concentration was measured by spectrophotometer at measuring length of 260 nm. Quantification of WSSV viral copy number in infected shrimp samples was conducted on family pool samples, using qPCR protocols as described by Jang et al. (2009). Briefly, q-PCR was performed using WSSV primer pair RT-WSSV-F154 (5′-CCA GTT CAG AAT CGG ACG TT-3′) and RT-WSSV-R154 (5′-AAA GAC GCC TAC CCT GTT GA-3′) amplified the fragment of 154 bp. The Taqman probe was synthesized and labeled with fluorescent dyes, 6-carboxyfloroscein (FAM, fluorophore on the 5′ end and N-[4-(4-dimethylamino) phenylazo] benzoic acid (DABCYL; quencher) on the 3′ end). The copy number of WSSV was calculated by comparing the threshold cycle value of samples against a standard curve generated using 10-fold diluted series of standard WSSV DNA concentration (Phuthaworn et al., 2016).

### Data Recording

Family information: Family origin and parental information were identified prior to tagging and the challenge test. The number of tested shrimp in each family and spawning time were recorded together with tagging code and their parents (sire and dam).

Body measurements: At tagging, 30 post-larvae (PL) shrimps were randomly chosen and individually measured of body weight using a digital balance (to nearest 0.1 g) and total length using a ruler before they were introduced into the infectivity tank system. In addition, siblings of the challenged test families reared in biosecurity tank systems were also measured of body traits at harvest (average weight of 24 g at about 150 days post-hatch).

Disease resistance data: Dead and moribund shrimps during the challenge test were collected every hour in the first three days. Subsequently the number of dead/moribund shrimps were recorded three times a day until the experiment was completed. Data recording was stopped when the population showed 50% mortality at about 15 days artificial post-test. In this study, WSSV resistance was measured as survival rate at different experimental periods. Survival traits in the challenge experiment were treated as binary variables in which dead shrimps were designated as zero, and alive animals at the end of the test were assigned a number one. The survival data was calculated for six different times during the challenge experiment, from the commencement of the test to day 3, 5, 7, 9, 12, and 15 (S1, S2, S3, S4, S5, and S6, respectively). Also note that in this study, survival was defined as the trait from the start of test until a defined time (i.e., 3, 5, 7, 9, 12, and 15 days post-test), and the survival traits of all periods included mortality at the start of the experiment.

### Statistical Analysis

#### Survival Rate and General Linear Model Analysis

Preliminary analysis using general linear model was firstly used to calculate basic statistics and identify survival trend in the high and low resistance families, using SPSS v.24 (Statistics, 2010). The viral copy number in infected shrimp samples was calculated by using qPCR method (Phuthaworn et al., 2016) and analyzed on software Rotor-Gene 2.1.0.9. Pathogenic differences as measured by the viral copy number (or viral load/ viral titer) between the high and low resistance families were examined using Kaplan–Meier estimator (SPSS v.24).

#### Genetic Parameters

To estimate the heritability for WSSV resistance, two different approaches were used (1) a linear mixed model (LMM) and (2) a threshold logistic generalized mixed model (TLGM) in ASREML 3.0 software (Gilmour et al., 2009).

The initial LMM model included the random effects of the additive genetics of individual shrimp and the common full-sib groups (c^2^) together with all possible fixed effects of spawning time and age from birth to the challenge test. The effect of spawning time was not statistically significant (*P* > 0.05) and not included in the final model. Logarithmic Likelihood Ratio Test (LRT) indicated that the random effects of common full-sib groups were not significant for WSSV resistance. Nevertheless, it was also included in the model to avoid any possible bias in genetic parameter estimates. The final LMM model is written in a matrix form as below:

[model 1], y=Xb+Za+Wc+e

where **y** is the vector of observations for resistant traits studied; **b** is the vector of the fixed effect (i.e., the linear co-variable of day from birth to the challenge test); **a** is the vector of the random sire and and(dam) effects that use a single matrix in the mixed model ∼ (0, **A**${\hat{\sigma }}_{s(d)}^{2}$) where **A** is the numerator relationship matrix estimated from the pedigree; **c** is the vector of common full-sib effects; and **e** is the vector of the random error term ∼ (0, **I**${\hat{\sigma }}_{e}^{2}$) where **I** is the identity matrix. **X, Z,** and **W** are the incidence matrices related to the fixed, random and common full-sib effects. A similar model to Equation 1 was also reported in previous studies (Krishna et al., 2011; Marjanovic et al., 2016; Nguyen et al., 2018). In ASReml, the “and(dam)” option used assumes equal variance of sire and dam (${\hat{\sigma }}_{s}^{2}={\hat{\sigma }}_{d}^{2}$).

Heritability for WSSV resistance was estimated from a single trait model and the correlation between traits studied were achieved from bivariate analyses. The bivariate model had the same fixed and random effects as the single trait Equation (1). In the model 1, heritability for resistance was calculated as *h*^2^ = $\frac{4{\hat{\sigma }}_{s}^{2}}{2{\hat{\sigma }}_{s}^{2}+{\hat{\sigma }}_{c}^{2}+{\hat{\sigma }}_{c}^{2}}$ where ${\hat{\sigma }}_{s}^{2}$ is the variance component of sire (${\hat{\sigma }}_{s}^{2}={\hat{\sigma }}_{d}^{2}$), ${\hat{\sigma }}_{c}^{2}$ is the common full-sib variance and ${\hat{\sigma }}_{e}^{2}$ is the environmental variance.

Phenotypic (*r_p_*) and genetic correlations (*r_g_*) between resistant traits (S1; S2… S6) were calculated as co-variance divided by the product of the standard deviations of traits: *r_p_* and/or *r_g_* = $\frac{{\hat{\sigma }}_{12}}{\sqrt{{\hat{\sigma }}_{1}^{2}\sqrt{{\hat{\sigma }}_{2}^{2}}}}$ where ${\hat{\sigma }}_{12}$ was the estimated additive genetic or phenotypic covariance between the two traits and ${\hat{\sigma }}_{1}^{2}$ and ${\hat{\sigma }}_{2}^{2}$ are the additive genetic or phenotypic variances of trait 1 and 2, respectively. However, only the genetic correlation between WSSV resistance and body weight was estimated because the traits were measured on different animals, specifically body weight was measured on siblings of the challenge test candidates. There was no environmental covariance between them and hence, the phenotypic correlation did not exist.

The threshold logistic generalized mixed models (TGMM) (model 2) had the same fixed and random effects as model 1. The model 2 presumed that the data followed a binomial distribution with a logit link functions $\hat{\text{p}}$ = *e^x^*/(1 + *e^x^*)), where *p* is the survival probability of individual shrimp recorded accumulatively at 3, 5, 7, 9, 12, and 15 days and *x* is a linear predictor. The co-variable effect was the same as those described in model 1 (**b** = the fixed effect of age, i.e., day from birth to the challenge test). The vector of the random effects consisted of the additive genetic effects of sire and dam (**a**), and common full-sibs, **c** and the vector of the error term **e**. **X, Z,** and **W** are the incidence matrices related to the fixed and random effects (Model 2):

[Model 2] $$\text{Log}\left(\frac{P}{1-P}\right)=\mathrm{Xb}+\mathrm{Za}+\mathrm{Wc}+e$$

Heritability under model 2 was calculated as h^2^_*o*_ = $\frac{4{\hat{\sigma }}_{s}^{2}}{2{\hat{\sigma }}_{s}^{2}+{\hat{\sigma }}_{c}^{2}+{\hat{\sigma }}_{e}^{2}\frac{\pi^{2}}{3}}$ where: ${\hat{\sigma }}_{s}^{2}$, ${\hat{\sigma }}_{c}^{2}$ and ${\hat{\sigma }}_{e}^{2}$ are the sire, common full-sib and residual variances, respectively.

As WSSV resistance was recorded as binomial observations, estimates of *h^2^* on the liability scale were transformed to the observed scale (0/1) using the formula of Robertson and Lerner (1949):

[3] $$h_{O}^{2}=h_{L}^{2}\frac{z^{2}}{p(1-p)}$$

where $h_{O}^{2}$ is the heritability on the observed (0/1) scale, $h_{L}^{2}$ is the estimated heritability on the liability (logit) scale, *p* is a proportion of a given survival rate in the data, and *z* is the height of the ordinate of normal distribution corresponding to a truncation point applied to *p* proportion of survival after the challenge test.

For completeness, sire model and animal model were also used to obtain heritability estimates for WSSV resistance (Supplementary Table S1).

## Results

### The Challenge Test

**FIGURE 1:**
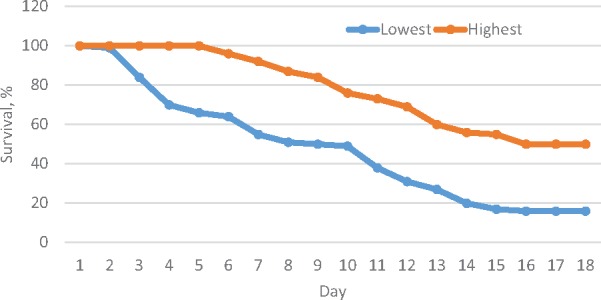
Survival trend over 15 days period after the challenge test in the highest and lowest resistance families.

Shrimp artificially infected with WSSV showed typical clinical signs of white spot syndrome disease. After 48 h infection, the tested shrimps showed apathetic behavior near the tank edge, slow movement, dis-orientation and body discoloration. The first deaths were recorded on the third day of infection (Figure 1). Histological tests on exoskeleton resulted in abnormal nuclei in enlargement of chromatin including eosinophilic inclusions in early phase infection and basophilic inclusions in the late stage infection. In addition, Cowdry A-type inclusions were displayed in intra-nuclei paralleled with a halophilic nuclear membrane.

### Pathogenic Screening

The concentration of viral DNA copies in moribund/or dead shrimp was examined in high and low resistant families against WSSV, using qPCR. There was a significant difference (*P* < 0.001) in WSSV viral load between the high resistant and low resistant families (Table 1). The amount of viral load was significantly lower in high than low resistant shrimp families, suggesting that resistance mechanism in shrimp was likely to diminish viral transmission and proliferation in the high resistant families.

**Table 1:** WSSV viral load (in log10 scale) in low resistant and high resistant families.

Group of families	Number of families	Number of tested shrimp	Viral load (copies μg^−1^ of DNA)	Maximum of viral load	Minimum of viral load
Low resistance	15	150	3.14 × 10^8a^	6.79 × 10^8^	6.4 × 10^7^
High resistance	15	150	1.15 × 10^8b^	3.24 × 10^8^	6.01 × 10^6^

*Different superscript letters in a column show statistical difference with P < 0.05.*

### Overall Survival Rate and Growth Data

In the challenge test period of 15 days, the mean survival rate of 150 full- and half-sib families was 42.8%, ranging from 16 to 50% (Table 2). Based on the analysis of three successive intervals, the lowest survival rate was about 84.6% in the period of day 3 and 5, while the highest value of 94.5% was observed during day 7–9 (Supplementary Figure F1).

**Table 2:** Number of observations (n), mean, standard deviation, minimum, and maximum survivals over six different experimental periods.

Trait	Unit	n	Mean	SD	Min	Max
**SURVIVAL RATE**						
S1	%	15,000	92.1	26.9	64	100
S2	%	15,000	81.0	39.2	51	100
S3	%	15,000	72.0	44.9	43	98
S4	%	15,000	66.5	47.2	31	95
S5	%	15,000	51.2	49.9	17	76
S6	%	15,000	42.8	49.5	16	50
**BODY TRAITS**						
Weight at tagging	g	4500	2.2	0.31	1	5.1
Length at tagging	mm	4500	63.5	2.90	46	88
Weight at harvest	g	5491	24.0	5.64	5.1	54.3
Length at harvest	cm	5491	15.0	1.11	1.5	19.8

*S1, S2, S3, S4, S5, and S6: Survival rate from the start of the experiment (0 day) to 3, 5, 7, nine, 12, and 15 days after the challenge test. Min = lowest survival family and Max = highest survival family.*

For growth traits (body weight and length) that were measured on individually tested shrimp prior to the experiment, the mean value was 2.2 ± 0.31 g and 63.5 ± 2.9 mm, respectively, after 15 days (Table 2). After 50 days of grow-out in biosecurity systems, their siblings reached 24.0 ± 5.64 g at time of harvest in December 2017.

### Family Variation in WSSV Resistance and Survival Trend in High and Low Resistant Families

There was significant variation (*P* < 0.001) in WSSV resistance among 150 challenged shrimp families over the entire experimental period (Figure 1). The greatest variation in the survival rate was observed in the period of day 9 and 12 (from 60 to 97%) (Supplementary Figure F1). A similar trend was also observed for S1–S5 (*P* < 0.05–0.01). For example, the survival rate ranged from 64 to 100% for S1.

**FIGURE 2:**
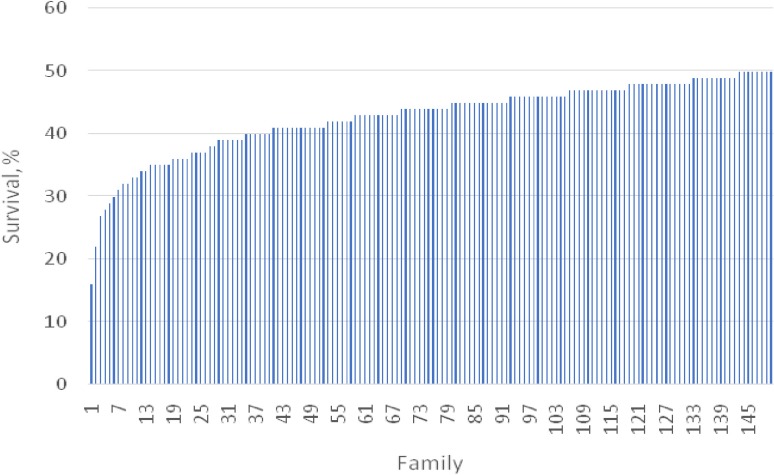
Variation in survival rate among 150 families involved in the challenge test in 15 days.

The survival trend also differed significantly (Kaplan–Meier estimator and log-rank test, *P* < 0.001) between the highest and lowest resistant families (Figure 2). In both groups, mortality did not occur in the first 2 days. After the challenge test, mortality occurred earlier and at a faster rate in the low than high resistance families (Supplementary Figure F2).

### Heritability and Common Environmental Effects

Heritability was estimated from two different statistical models: LMM (model 1) and logistic threshold mixed model (model 2). Across the models, heritability for WSSV resistance in this shrimp population was moderate in the early experimental periods (S1–S4), while a low h^2^ estimate was observed 12 and 15 days after the challenge test (Table 3). The value of heritability estimated for day 12th and 15th from both models were close to zero (the h^2^ = 0.06 ± 0.02 and 0.01 ± 0.01, respectively).

**Table 3:** Heritability (h^2^ ± s.e.) for WSSV resistance, and h^2^ and common full-sib effects (c^2^ ± s.e.) for body traits.

Trait	Model 1		Model 2	Transformed heritability	
Survival rate	h^2^	c^2^	h^2^	h^2^_*T*_	c^2^
S1	0.38 ± 0.07	0.013 ± 0.003	n.e.	n.e	n.e.
S2	0.28 ± 0.05	0.023 ± 0.005	0.64 ± 0.11	0.31 ± 0.05	0.030 ± 0.010
S3	0.18 ± 0.04	0.009 ± 0.002	0.31 ± 0.07	0.17 ± 0.04	0.015 ± 0.004
S4	0.14 ± 0.03	0.008 ± 0.002	0.20 ± 0.05	0.12 ± 0.03	0.012 ± 0.003
S5	0.05 ± 0.02	0.002 ± 0.001	0.06 ± 0.02	0.04 ± 0.01	0.002 ± 0.001
S6	0.01 ± 0.01	0.000 ± 0.000	0.01 ± 0.01	0.01 ± 0.01	0.000 ± 0.000
**Body traits**	**h^2^**	**c^2^**			
Weight at tagging	0.45 ± 0.08	n.e.			
Length at tagging	0.50 ± 0.07	n.e.			
Weight at harvest	0.09 ± 0.03	0.02 ± 0.01			
Length at harvest	0.10 ± 0.03	0.02 ± 0.01			

*S1, S2, S3, S4, S5, and S6: Survival rate from the commencement of the experiment (0 day) to 3, 5, 7, 9, 12, and 15 days after the challenge test, n.e., not-estimable. Model 1 = Linear mixed model and Model 2 = Threshold logistic mixed model. The transformed heritability was obtained from Equation 3. The LMM used to analyze body traits included both the additive genetic and common full-sib effects. Heritability (h^2^) and common full-sib estimates (c^2^) for body traits were calculated as:*$h^{2}=\frac{{\hat{\sigma }}_{a}^{2}}{{\hat{\sigma }}_{a}^{2}+{\hat{\sigma }}_{c}^{2}+{\hat{\sigma }}_{e}^{2}}$*and*$c^{2}=\frac{{\hat{\sigma }}_{c}^{2}}{{\hat{\sigma }}_{a}^{2}+{\hat{\sigma }}_{c}^{2}+{\hat{\sigma }}_{e}^{2}}$*, where*${\hat{\sigma }}_{a}^{2}$*= the additive genetic variance,*${\hat{\sigma }}_{c}^{2}$*= common full-sib variance and*${\hat{\sigma }}_{e}^{2}$*= residual variance.*

When the heritabilities obtained for WSSV resistance on the liability scale (logit) were back-transformed, using equation 3, the transformed heritability estimates ranged from 1 to 31% (Table 3).

The heritability for body weight and total length measured prior to the challenge test were moderate (0.45 ± 0.08 and 0.50 ± 0.07, respectively). However, those obtained for body traits recorded on animals that were not challenge tested, were low (0.09 and 0.10 ± 0.03, respectively).

The common full-sib effect (c^2^) based on logarithmic LRT was not significant for WSSV resistance in this study. The LogL value did not change when the full or reduced model was used. The c^2^ effect explained less than 3% of total variance for WSSV resistance and only 2% of the total variance for body traits in this population (Table 3).

### Phenotypic and Genetic Correlations

The estimates of phenotypic and genetic correlations between survival traits are shown in Table 4. The phenotypic correlations ranged from 0.31 ± 0.01 to 0.85 ± 0.01. The genetic correlations were high and approaching one (0.80 ± 0.05–0.97 ± 0.01). Both phenotypic and genetic correlations between successive challenge phases were greater than those that were further apart. As described above, all the survival traits examined here included mortality of previous periods and hence, the phenotypic and genetic correlations should be interpreted in this context.

**Table 4:** Phenotypic (above) and genetic (below the diagonal) correlation among survival traits.

Traits	S1	S2	S3	S4	S5	S6
S1		0.63 ± 0.01	0.50 ± 0.01	0.44 ± 0.01	0.31 ± 0.01	0.27 ± 0.01
S2	0.89 ± 0.03		0.79 ± 0.05	0.69 ± 0.01	0.50 ± 0.01	0.43 ± 0.01
S3	0.82 ± 0.04	0.97 ± 0.01		0.88 ± 0.01	0.64 ± 0.01	0.54 ± 0.01
S4	0.80 ± 0.05	0.94 ± 0.02	0.98 ± 0.01		0.72 ± 0.01	0.61 ± 0.01
S5	0.82 ± 0.06	0.89 ± 0.04	0.90 ± 0.04	0.88 ± 0.04		0.85 ± 0.01
S6	n.e.	n.e.	0.98 ± 0.11	0.92 ± 0.12	0.99 ± 0.05	

*S1, S2, S3, S4, S5, and S6: Survival rate from the commencement of the experiment (0 day) to 3, 5, 7, 9, 12, and 15 days after the challenge test.*

Table 5 presents genetic correlations between the survival traits and body weight prior to the challenge test (about 2 g) and at harvest (24 g). All the estimates were negative and significant. Two exceptions were the positive genetic correlation between body weight and S5 or between body weight and S6, however, the estimates were not different from zero due to their high standard errors.

**Table 5:** Genetic (r_*G*_) correlation between body weight and survival rate.

Traits	Weight at harvest	Weight prior to the experiment
S1	−0.49 ± 0.12	−0.17 ± 0.07
S2	−0.56 ± 0.11	−0.22 ± 0.07
S3	−0.59 ± 0.10	−0.17 ± 0.08
S4	−0.63 ± 0.10	−0.15 ± 0.08
S5	−0.47 ± 0.14	0.13 ± 0.10
S6	−0.33 ± 0.29	0.40 ± 0.21

*S1, S2, S3, S4, S5, and S6: Survival rate from the commencement of the experiment (0 day) to 3, 5, 7, 9, 12, and 15 days after the challenge test.*

## Discussion

### Heritability for WSSV Resistance

In this study, we report, for the first time, genetic parameters for WSSV resistance and body weight at six different infection phases in juvenile shrimp. The heritability estimates for survival rate in the first three measurements were higher than those obtained in the later periods (0.36–0.20 for 3 days, 5 days vs. 0.04–0.14 for 9, 12, and 15 days). This decelerating trend of heritability was also observed when statistical analysis was conducted separately for six different time intervals from day 1 to 3 (S1–3), 3–5 (S3–5) and 12–15 (S12–15) (results not tabulated). The close to zero heritabilities for 12 and 15 days were likely because mortalities had ceased before 15 days of infection and hence, the resistance to WSSV may have been less dependent on the innate immunity differences between the animals. This likely relied more on non-genetic factors (including re-exposure or other random non-genetic stress factors). These non-genetic factors could have created “background noise” masking genetic effects on WSSV resistance in the later experimental periods. The finding of our study is in line with the results reported by Ødegård et al. (2011) that selection for TSV resistance at an average size of 1 g shrimp improved farm survival of *L. vannamei* against TSV epizootics. Depending on virulence, challenge method, environmental condition and cut-off for end of experiment, our results obtained here suggest that selection for WSSV resistant trait can be effective in the early phase of infection in *L. vannamei*.

Earlier studies reported heritability for WSSV resistance during a grow-out phase in Whiteleg shrimp. Caballero-Zamora et al. (2015) showed that the heritability for WSSV resistance to a natural disease outbreak in *L. vannamei* reared in ponds to harvestable size was low (0.06 and 0.02). A similar estimate was obtained in a different shrimp population (Bishop and Woolliams, 2010; Caballero-Zamora et al., 2015). In other reports (Gitterle et al., 2005, 2006a,b), the heritability for WSSV resistance in adult shrimp populations was low (<10%). The higher estimate of heritability for WSSV resistance in our study especially during the first five days of infection than the published information can be explained because our experiment was carried out during the juvenile rearing stage. On the other hand, other studies in grow-out shrimps under natural disease outbreak or field experiment were largely influenced by environmental factors, as well as unassembled recording, inaccurate diagnosis or changeable exposure (Bishop and Woolliams, 2010).

Across the aquaculture species, the heritability estimated for pathogenic resistance from challenge test experiments is moderate to high, such as for TSV resistance in *L. vannamei* (Argue et al., 2002) (h^2^ = 0.28 ± 0.14), vibrio and nervous necrosis virus in Atlantic cod (Bangera et al., 2011) (h^2^ = 0.16 ± 0.04–0.68 ± 0.14) and *Aeromonas salmonicida* resistance in Atlantic salmon (Gjerde et al., 2009) (0.46 ± 0.13). Our estimate of heritability for WSSV resistance in Whiteleg shrimp was lower than the results reported in the literature for other pathogenic agents. The existence of the heritable genetic variation during the early phase of infection indicates that selection for improved WSSV resistance in Whiteleg shrimp can achieve genetic response.

### Genetic Correlations

Genetic association between body weight and resistance to WSSV in *L. vannamei* at different infection times during the challenge test has not yet been reported. In our study, the genetic correlations (*r_g_*) between WSSV resistance and body weight at tagging (∼2 g) and harvest (24 g) were negative in the early phase of rearing but the *r_g_* estimates were not significant between body weight and WSSV resistance recorded over the entire experimental period (0–15 days). Although moribund/dead shrimps were collected frequently, cannibalism may have occurred due to size variation in the population. Bigger shrimp likely had greater exposure to the pathogen during the challenge test. Collectively, it is suggested that selection for high growth may not have detrimental effect on disease risk of the population. Selection for high growth will reduce production time and hence, reducing exposure to the pathogen and risk of infection. However, when more data is accumulated, further analysis should be conducted to confirm the genetic correlations between WSSV resistance and body weights in this population of *L. vannamei*.

Furthermore, the non-significant genetic correlations between S5 or S6 and body weight in our study also suggest that the genetic relationships between growth and survival were growth stage-specific. This is in line with previous reports in *L. vannamei* and fish species. In the study of Caballero-Zamora et al. (2015), none of the genetic correlation estimates between BW and survival were significantly different from zero (−0.98 ± 0.57). However, there are reports of negative (i.e., unfavorable) genetic correlations between body weight and WSSV (−0.55 ± 0.18 to −0.64 ± 0.19) (Gitterle et al., 2005, 2006a) or between body weight and TSV resistance (−0.46 ± 0.18) in *L. vannamei* (Argue et al., 2002) and between weight and viral hemorrhagic septicemia in rainbow trout (−0.14 to −0.33) (Henryon et al., 2002). Our results together with those reported in the literature suggest that it is necessary to examine the genetic relationships between growth and disease resistance on individual case basis to assist the design and conduct of genetic improvement programs for aquaculture species in question.

### Common Environmental Effect

Our analysis using a full mixed model showed that the common full-sib effects (c^2^) were not significant for WSSV resistance. Earlier studies (Gitterle et al., 2005) did not report the c^2^ effects for survival. Generally, survival of *L. vannamei* is closely related to management practices and rearing conditions, such as water temperature (Li et al., 2015), rearing condition and stocking density and feeding (Moss et al., 2012; Sui et al., 2016). In the present study, environmental effects common to full-sibs for WSSV resistance were not significant, indicating that a standardized hatchery and management practices from breeding to rearing and tagging were applied to all the families produced. However, when more data is accumulated, the maternal and c^2^ effects should be included in the statistical model to avoid possible biases in the genetic parameter estimates. With the mating system of one male to two females used in this study, it is difficult to separate maternal from common environmental effects. Minimizing the maternal and c^2^ effects can help achieve greater genetic gain for WSSV resistance and body weight in future breeding programs for this species.

### Practical Implications and Significance for Breeding Programs

With the growing complication of diseases on commercial production of *L. vannamei*, development of disease resistant varieties is considered as an effective solution in disease prevention. In this study, we showed that regardless of statistical models used, there is substantial additive genetic variation in WSSV resistance especially during the early phase of the challenge test that provides prospects for future improvement of this trait in the present population of Whiteleg shrimp (Supplementary Table S1). The heritability for WSSV was moderate in the early phase of the experiment but low in the later stage of the challenge test, suggesting that selection can be conducted during the juvenile stage to minimize production costs, shortening the selective cycle, and thereby increasing the rate of genetic gain achieved for WSSV resistance. However, also note that the high genetic correlations between WSSV resistance at successive test periods were partially attributed to part-whole relationships of the data as described in section Data Recording.

This study developed standardized experimental protocols to accurately determine survival rate during the challenge test and the viral copy number of breeding families. They are useful reference sources to apply to other crustacean species. A large number of full- and half-sib families produced in this study and the significant variation in WSSV resistance among the families provide scope for intense selection to increase the rate of genetic improvement for WSSV resistance in Whiteleg shrimp.

## Conclusion

WSSV resistance is heritable in this population of *L. vannamei*, suggesting that genetic improvement in WSSV resistance can be achieved via selective breeding. Genetic correlations of WSSV resistance between different infection times were high and hence, selection can be practiced early, e.g., 3–5 days after challenge test to save costs associated with feeding and rearing; thereby increasing the efficiency of selective breeding programs. Due to a single generation data records for traits examined here, continuing collection of more data is needed to better understand genetic relationships between body weight, viral titer and WSSV resistance in the present population of Whiteleg shrimp. Examine the effect of genotype by environment interaction on these traits also merits further study to assist the design and conduct of genetic improvement programs for *L. vannamei*.

## Ethics Statement

Animal Ethics approval was not required for crustacean species. All the experimental procedures were conducted in accordance with standard commercial practices and regulations of the local government.

## Author Contributions

TTT, NHH, NHuN, and NHoN conceived, designed and conducted the study, analyzed the data and prepared the manuscript. WK read and provided comments. All the authors approved the submission.

## Conflict of Interest Statement

The authors declare that the research was conducted in the absence of any commercial or financial relationships that could be construed as a potential conflict of interest.
