# Supplementary material for: Genetic Variation in Disease Resistance Against White Spot Syndrome Virus (WSSV) in Liptopenaeus vannamei
Source: Front Genet. 2019 Mar 28;10:264. doi: 10.3389/fgene.2019.00264 (PMC6447704; doi:10.3389/fgene.2019.00264)
Supplement: Supplementary file 1 [file Data_Sheet_1.zip › Supplementary Files 3/Supplementary Figure F1.docx]

Supplementary Figure F1: Example of mortality variation among 150 families in two intervals (7-9d, high mortality vs. 9-12d, low mortality).
