# Supplementary material for: Genetic Variation in Disease Resistance Against White Spot Syndrome Virus (WSSV) in Liptopenaeus vannamei
Source: Front Genet. 2019 Mar 28;10:264. doi: 10.3389/fgene.2019.00264 (PMC6447704; doi:10.3389/fgene.2019.00264)
Supplement: Supplementary file 1 [file Data_Sheet_1.zip › Supplementary Files 3/Supplementary Figure F2.docx]

|  |  |
| --- | --- |
| Supplementary Figure F2: Survival trend in three highest and lowest survival families over the entire experimental period of 15 days (L2 and L3: The second and third lowest resistance families; H2 and H3 = The second and third highest resistance families) | |
