# Supplementary material for: Genetic Variation in Disease Resistance Against White Spot Syndrome Virus (WSSV) in Liptopenaeus vannamei
Source: Front Genet. 2019 Mar 28;10:264. doi: 10.3389/fgene.2019.00264 (PMC6447704; doi:10.3389/fgene.2019.00264)
Supplement: Supplementary file 1 [file Data_Sheet_1.zip › Supplementary Files 3/Supplementary Table S1.docx]

Supplementary Table S1: Heritability (±s.e.) for WSSV resistance estimated from sire or animal model

| Trait | Sire model | |  | Animal model | |
| --- | --- | --- | --- | --- | --- |
|  | LMM | TLGM |  | LMM | TLGM |
| S1 | 0.26 ± 0.08 | n.e. |  | 0.30 ± 0.05 | 0.36 ± 0.02 |
| S2 | 0.17 ± 0.04 | 0.43 ± 0.09 |  | 0.25 ± 0.04 | 0.20 ± 0.02 |
| S3 | 0.12 ± 0.03 | 0.21 ± 0.05 |  | 0.16 ± 0.03 | 0.13 ± 0.02 |
| S4 | 0.10 ± 0.02 | 0.14 ± 0.03 |  | 0.14 ± 0.02 | 0.11 ± 0.01 |
| S5 | 0.04 ± 0.01 | 0.05 ± 0.01 |  | 0.04 ± 0.01 | 0.04 ± 0.01 |
| S6 | 0.01 ± 0.01 | 0.01 ± 0.01 |  | 0.01 ± 0.01 | 0.01 ± 0.01 |

S1, S2, S3, S4, S5 and S6: Survival rate from the start of the experiment (0 day) to three, five, seven, nine, twelve and fifteen days after the challenge test

n.e. = out of the parameter space

LMM = Linear Mixed Model and TLGM = Threshold Logistic Generalised Model
